# Coronavirus Disease and the Shared Emotion of Blaming Others: Reviewing Media Opinion Polls During the Pandemic

**DOI:** 10.2188/jea.JE20210169

**Published:** 2021-07-05

**Authors:** Yusuke Inoue, Taketoshi Okita

**Affiliations:** 1 Department of Public Policy, The Institute of Medical Science, The University of Tokyo, Tokyo, Japan; 2 Department of Medical Ethics, Tohoku University Graduate School of Medicine, Sendai, Japan

In Japan, the revised Infectious Diseases Control Law^1^ and other amending acts, passed in February 2021, newly stipulates administrative penalties for those who refuse hospitalization and testing and those who do not comply with shortened business hours when required. Considering that Japan’s countermeasures against infectious diseases rely on individual voluntary behavioral changes, this revision of the law may become one of the major turning points for such countermeasures. Introducing penalties as a response to a pandemic should be considered with great caution. Individual behaviors are not determined solely by personal preferences,^2^ and harsh criticisms against individuals deemed non-compliant by the general population can lead to the alienation of patients and infected individuals and be inappropriate for infectious disease control.^3^

Therefore, we agree with the Japan Epidemiological Association,^4^ which states that great care must be taken when asking individuals to take excessive responsibility as a countermeasure against infectious diseases. Nevertheless, during the coronavirus disease (COVID-19) pandemic, frequent cases of individuals defaming those who acted differently and denouncing infected persons were observed in Japan.^5^ Studying how societal emotions are affected by individual responsibilities and their dynamics is important, especially when supporting or guiding individuals unable to comply with countermeasures against infectious diseases. It is also important for epidemiologists to understand the punitive emotions that allow the legalization of penalties for controlling diseases to gain lessons for future pandemics.

On the other hand, in the case of the current pandemic, especially when the situation is progressing rapidly and dynamically, it is difficult to conduct well-designed academic surveys to understand the dynamics of people’s emotions in a timely manner. Hence, we tried to infer the reactions of the public based on existing opinion polls, while paying attention to question content consistency and methodology of them.

We collected data from opinion polls conducted by major Japanese media firms from January 2020 to January 2021, including questions related to the introduction of penalties as countermeasures against COVID-19, as well as information on the survey period, method, and target population. These 12 opinion polls were conducted during the following periods: Yomiuri-NNN: April (a) and June (c) 2020, January (g, m) 2021; TBS-JNN: May (b) 2020, January (f) 2021; Asahi: November (d, e) 2020, January (l, q) 2021; NHK: January (i) 2021; Kyodo: January (h) 2021; Mainichi-SSRC: January (n) 2021; ANN: January (j, o) 2021; and Fuji-Sankei: January (k, p) 2021. The average number of respondents in each survey was 1,441 (minimum: 520; maximum: 2,187). The survey periods were largely divided into April to June 2020 (first phase), November to early December 2020 (second phase), and January 2021 (third phase). The first phase included the period from the declaration of the state of emergency in Japan, the first wave of COVID-19 cases (April 2020), and the end of the state of emergency (June 2020). Thereafter, in Japan, the number of COVID-19 cases increased to more than double by August (the second wave); however, opinion polls were not conducted during this period. The second phase encompassed the beginning of the third wave. The third phase corresponded to when the number of cases continued to increase, and the second state of emergency was declared.

From these opinion polls, we identified a total of 17 questions related to respondents’ approval or disapproval of introducing penalties. The details of each question were obtained from Yomiuri-NNN,^6^ TBS-JNN,^7^ Asahi,^8^ NHK,^9^ Mainichi-SSRC,^10^ ANN,^11^ and Fuji-Sankei.^12^ Kyodo News reported the survey methods in related newspapers (eg, *Shimotsuke Shinbun*^13^), and detailed questions were obtained via direct inquiry (January 26, 2021). Among the questions, 12 related to restrictions on the actions of citizens and businesses (eTable 1), and 5 to infected persons (eTable 2). The responses regarding the introduction of penalties were categorized into “positive” and “negative” and arranged chronologically. We also reviewed the clarity of the questions, the neutrality of the choices, and the transparency of the respondent information.

As a result of the review, we identified two characteristics related to the transition of people’s punitive emotions. First, trends in supporting the introduction of penalties were not always proportional to the number of cases (Figure 1). According to the results of surveys “a” and “b”, public support for penalizing residents and businesses that did not actively cooperate with stay-at-home requests peaked in April and May 2020. The rise in punitive emotions during this period can be understood as a stern view against individuals who do not follow common public health measures related to unknown infectious diseases.

**Figure 1.**
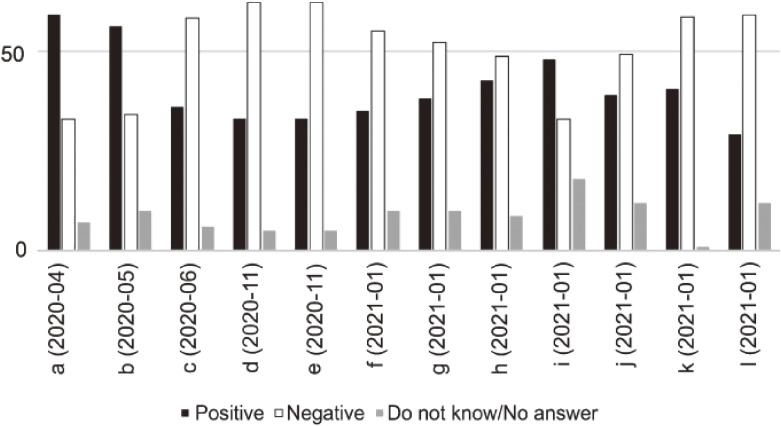
Responses to questions on introducing penalties for non-cooperation with stay-at-home requests Note: The values are in percentages (%). The abbreviations concerning the questions of the opinion polls and their timing of implementations are as follows: “a” (Yomiuri-NNN, April 2020), “b” (TBS-JNN, May 2020), “c” (Yomiuri-NNN, June 2020), “d” (Asahi, November 2020), “e” (Asahi, November 2020), “f” (TBS-JNN, January 2021), “g” (Yomiuri-NNN, January 2021), “h” (Kyodo, January 2021), “i” (NHK, January 2021), “j” (ANN, January 2021), “k” (Fuji-Sankei, January 2021), “l” (Asahi, January 2021).

After this peak, the trend had changed dramatically, as demonstrated in the opposite results of “a” and “c” of polls conducted by the same media firm using the same questions. According to polls “c” onward, support did not constitute a majority, excluding “i”. In addition to an increase in the cumulative knowledge and experience of the pathogen causing COVID-19, there was also a likely increase in negative opinions about the resolutions that could be attained through punishment, as more people became aware of the high price of perpetual self-restraint. Concerning “i”, which revealed peaked support for penalties, it should be noted that the question in the survey also mentioned the possibilities of “financial support for businesses” in return of cooperation with self-constraint requests. Introduction of such compensation may have made some respondents of this question become receptive to the introduction of penalties (Similar phrases were also contained in “h” and “k”). Nevertheless, the fact that approximately 30% of poll respondents still favored penalizing problematic behavior should not be also ignored.

Second, trends in supporting penalizing non-compliance of infected persons differed from the abovementioned reactions regarding non-cooperation with stay-at-home requests (Figure 2). This survey item appeared after January 2021 (“m” to “q”), when positive opinions about the introduction of penalties were dominating. It highlighted a different aspect of punitive emotions compared with those during early spring (the first wave of COVID-19 cases). Although there have been occasional reports of behavioral problems among infected individuals, the causal relationship between this and the spread of infection has not been proven. It is necessary to further examine the underlying factors associated with an increase in punitive emotions. Many emotional factors may be considered, such as anxiety about the medical crisis that became evident in autumn, when the third wave of COVID-19 cases began, and sympathy for medical staff who are exhausted and anxious about their health. Additionally, an element of “victim-blaming”^14^ was evident among discussions, which solely focused on the violation of societal rules without taking into account the circumstances of the behaviors of infected individuals.

**Figure 2.**
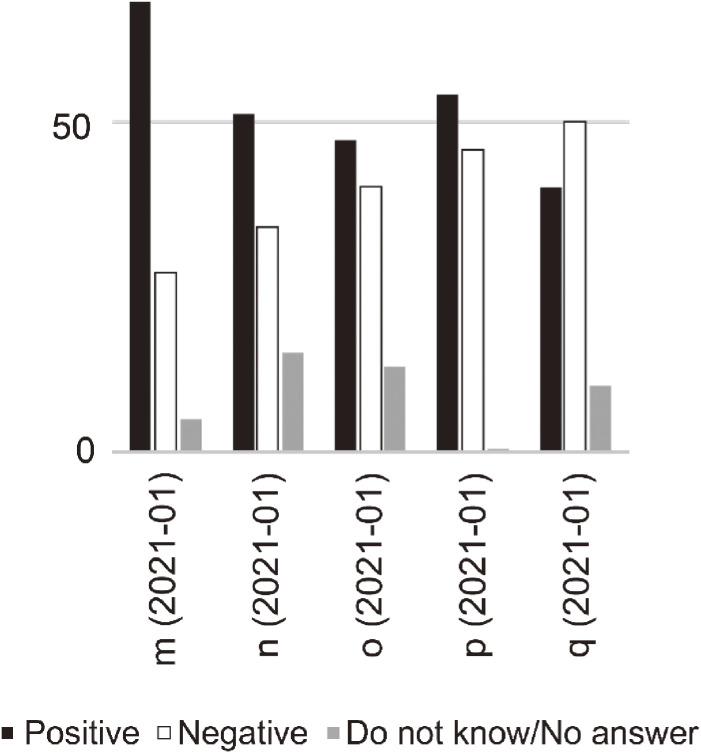
Responses to questions on introducing penalties for the non-compliant behaviors of infected persons Note: The values are in percentages (%). The abbreviations concerning the questions of the opinion polls and their timing of implementations are as follows: “m” (Yomiuri-NNN, January 2021), “n” (Mainichi-SSRC, January 2021), “o” (ANN, January 2021), “p” (Fuji-Sankei, January 2021), “q” (Asahi, January 2021).

Adding to these two results, we must also note irregularities among these surveys. Some of the questions contained double-barreled elements. Notably, the question in survey “n” contained mixed elements, possibly increasing cautious opinions about the introduction of penalties. The results of the question in survey “q” must be interpreted carefully, since it concerned respondents’ approval or disapproval of “imprisonment” rather than the acceptability of penalties. Moreover, there were surveys in which the respondents were strongly prompted to choose “approval” or “disapproval” about the questions, with no option to say, “I do not know” or “I have no answer here” (as was available in surveys “k” and “p”). Such questions could have resulted in the small number of “Do not know” and “No answer” responses, irrespective of the possibility that some people found it difficult to answer these questions with clarity. The partial inconsistency in poll’s theme should be also noted.

As seen in our results, there were fluctuations in the survey results. Therefore, the significance of the survey results at any given point in time is limited and cannot be generalized. The media should not arbitrarily report on previous survey results, and the limits of using the results of these polls should be clearly stated in newspaper articles that print them. The Nihon Shinbun Kyokai (Japan Newspaper Publishers & Editors Association) and JBA (The Japan Commercial Broadcasters Association) issued a joint statement^15^ on the issue of discrimination and prejudice toward individuals infected with COVID-19 in May 2020, stating that they will not condone human rights violations against them and will take action to curb the sensationalism seen in the news. However, the specific efforts each company plans to make remain unclear.

In summary, opinion polls can provide partial clues regarding trends in people’s punitive emotions when results from multiple firms are examined as a cluster. Epidemiologists should also consider the impact of poll results on people’s punitive emotions.

## APPENDIX A. SUPPLEMENTARY DATA

eTable 1. Questions on introducing penalties for non-cooperation with stay-at-home requests

eTable 2. Questions on introducing penalties for the non-compliant behaviors of infected persons and/or patients
