# Supplementary material for: Coronavirus Disease and the Shared Emotion of Blaming Others: Reviewing Media Opinion Polls During the Pandemic
Source: J Epidemiol. 2021 Jul 5;31(7):453–5. doi: 10.2188/jea.JE20210169 (PMC8187613; doi:10.2188/jea.JE20210169)
Supplement: Supplementary file 1 [file je-31-453-s001.pdf]

eTable 1. Questions on introducing penalties for non-cooperation with stay-at-home requests

Poll*	Question	Necessity of Penalties	Method/Number of Responses
a	Unlike Western countries, the state of emergency declared by the government does not have the compelling force to prohibit residents from going out. The procedure is to firmly request residents to stay at home. Do you think the request to stay at home is enough or not enough?	Enough (Disagree)<br>Not enough (Agree)<br>No answer	Telephone survey (n=1,176)
b	Regarding the special measures law on COVID-19, there have been growing calls to revise the law to introduce penalties for those who disregard the requests to stay at home. Are you in favor of or against such an amendment?	In favor (Agree)<br>Not in favor (Disagree)<br>No answer /Not sure	Telephone survey (n=2,187)
c	Same as Question "a".	Same as the options in Question "a"	Telephone survey (n=1,102)
d	Which is closer to your opinion about the measures against COVID-19, A or B? [A] Bars and restaurants that	A (Agree)<br>B (Disagree)	Mail survey (n=2,126)

	do not follow the instructions to shut down should be fined. [B] Bars and restaurants should be allowed to decide whether to shut down without being forced by the government.		
e	Which is closer to your opinion about the measures against COVID-19, A or B? [A] Nationals who do not comply with stay-at-home regulations should be fined. [B] Nationals should be allowed to decide whether to stay at home, without being forced by the government.	A (Agree)<br>B (Disagree)	Mail survey<br>(n=2,126)
f	The government, along with all parties, is working to revise the special measures law on COVID-19, considering whether to impose penalties on businesses, such as restaurants, that do not respond to the requests to shorten their business hours. Are you in favor of or against fines?	In favor (Agree)<br>Not in favor (Disagree)<br>No answer	Telephone survey<br>(n=2,143)
g	Do you agree or disagree with the provision of the bill to penalize bars and restaurants in the areas under a state of emergency for not responding to prefecture governors'	Agree<br>Disagree	Telephone survey<br>(n=1,093)

	orders to shorten business hours or shut down?		
h	Prime Minister Yoshihide Suga is considering offering compensation to bars and restaurants to support shortened business hours and closures in areas under the state of emergency, while also considering a bill to penalize those bars and restaurants that do not comply with these requests. Do you agree or disagree with the introduction of such a penalty?	Agree<br>Disagree<br>No answer / Not sure	Telephone survey (n=520)
i	The government is aiming to revise the special measures law on COVID-19 by combining financial support and penalties for businesses. Are you in favor of or against specifying penalties?	In favor (Agree)<br>Not in favor (Disagree)<br>No answer	Mail survey (n=1,278)
j	Under the state of emergency, penalties are being considered for businesses that do not respond to requests to shorten their business hours or shut down. Do you think it is necessary to amend the special measures law on COVID-19 and impose penalties?	Necessary (Agree)<br>Not necessary (Disagree)<br>Not sure, No answer	Telephone survey (n=1,842)

k	Do you agree or disagree with the government's introduction of penalties in combination with payouts related to requests for bars and restaurants to shorten their business hours or shut down in areas under the state of emergency?	Agree<br>Disagree	Telephone survey (n=1,104)
l	Do you agree or disagree with penalizing stores that do not comply with orders to shorten their business hours and shut down in areas under a state of emergency in the form of a fine of up to 500,000 yen?	Agree<br>Disagree	Telephone survey (n=1,647)

* The abbreviations concerning the questions of the opinion polls and their timing of implementations were as follows: "a" (Yomiuri-NNN, April 2020), "b" (TBS-JNN, May 2020), "c" (Yomiuri-NNN, June 2020), "d" (Asahi, November 2020), "e" (Asahi, November 2020), "f" (TBS-JNN, January 2021), "g" (Yomiuri-NNN, January 2021), "h" (Kyodo, January 2021), "i" (NHK, January 2021), "j" (ANN, January 2021), "k" (Fuji-Sankei, January 2021), "l" (Asahi, January 2021).

eTable 2. Questions on introducing penalties for the non-compliant behaviors of infected persons and/or patients

Poll*	Question	Necessity of Penalties	Method/Number of Responses
m	Do you agree or disagree with penalties for people infected with COVID-19 who refuse to be admitted to hospitals or those who do not respond to an inquiry from a health center?	Agree<br>Disagree	Telephone survey (n=1,093)
n	The government is considering penalties for businesses that refuse to shorten their hours and for infected people who refuse to be admitted to hospitals. Do you think these penalties are necessary?	Necessary (Agree)<br>Not necessary (Disagree)<br>Not sure	Telephone survey (n=1,079)
o	A proposal to amend the Infectious Diseases Prevention Act is under consideration that would impose criminal penalties, such as fines and imprisonment for up to 1 year, on persons infected with COVID-19 who refuse to be admitted to hospitals. Do you think the law should or	Necessary (Agree)<br>Not necessary (Disagree)<br>Not sure	Telephone survey (n=1,842)

	should not be amended?		
p	Do you agree or disagree with the policy of penalizing infected people who refuse to be admitted to hospitals?	Agree<br>Disagree	Telephone survey (n=1,104)
q	Do you agree or disagree with imposing up to 1 year of imprisonment or a fine of up to 1 million yen for people infected with COVID-19 who refuse to be hospitalized?	Agree<br>Disagree	Telephone survey (n=1,647)

* The abbreviations concerning the questions of the opinion polls and their timing of implementations were as follows; "m" (Yomiuri-NNN, January 2021), "n"(Mainichi-SSRC, January 2021), "o"(ANN, January 2021), "p" (Fuji-Sankei, January 2021), "q"(Asahi, January 2021).
